# Chains of extraction: shifting bioeconomies in India and East Africa

**DOI:** 10.3389/fsoc.2024.1149368

**Published:** 2024-07-26

**Authors:** Johanna Gondouin, Åsa Eriksson, Suruchi Thapar-Björkert

**Affiliations:** ^1^Department of Ethnology, History of Religions and Gender Studies, Stockholm University and Mångkulturellt Centrum, Stockholm, Sweden; ^2^Department of Government, Uppsala University, Uppsala, Sweden

**Keywords:** India, East Africa (Kenya), surrogacy, biocapitalism, colonial histories, globalization - economic development

## Abstract

Since the early 2000s, India has been a world leading hub for cross border reproductive treatments, in particular surrogacy, with the nation positioning itself as the “mother destination” for transnational commercial surrogacy, offering “First world services at Third world prices”. State policies, lack of legal regulation, state of the art medical infrastructure and a steady supply of women ready to take on the role as surrogate mothers against meager remuneration have been key factors behind the Indian success story. Yet, a gradual process of regulation in recent years, culminating in the introduction of the Surrogacy (Regulation) Bill 2020, has forced the industry to reinvent itself in order to maintain its role as a market leader in a booming global bioeconomy. This article takes the 2020 bill as a starting point for an exploration of the key trajectories that the Indian reproductive industry has taken since. This includes moving into new market segments, such as the unregulated practice of oocyte donation, and expanding globally into new geo-political contexts. Through these practices, India has successfully rebranded itself as a world leading “pre-conception assemblage hub” where embryos are assembled and implanted into surrogates who carry their pregnancies to term in countries with no protective legislation. The article begins to map the emerging links between the reproductive industry in India and East Africa - where diasporic networks are mobilized in the creation of new reproductive markets, dominated by Indian IVF providers. In particular, we discuss the current expansion in Kenya, which we situate against the backdrop of the colonial entanglements between the two countries. While the ART industry in Kenya is still young, we suggest that these emerging developments illuminate the effect of the ban on commercial surrogacy in India, which appears to have resulted in a partial relocation to countries that lack regulation, shifting the precarious conditions of surrogates in India to other women, elsewhere, in ways that rearticulate colonial racial hierarchies and migration patterns.

## Introduction

Transnational commercial surrogacy is part of a rapidly expanding global market in body parts and reproductive labor through which the body has been transformed into a commodity in an unprecedented way (Cohen, 2001; Scheper-Hughes, 2004; Sunder Rajan, 2007). Female reproductive biology is a main generative site in this bioeconomy, through the expansion of assisted reproductive technologies (ARTs) and new stem cell industries which are dependent on high volumes of human embryos, oocytes, fetal tissue and umbilical cord blood (Cooper and Waldby, 2008, 2014). Cooper and Waldby (2014) find that these oocyte and surrogacy markets point toward a gendered form of trade which is positioned on creating surplus value from women’s bodies. Importantly, it enables privileged women to make choices through the bodies of less privileged women; reproduction is stratified (Colen, 1995) according to hierarchies of class, race, ethnicity, nationality, migration status, religion and caste, creating an “international division of reproductive labor” (Parreñas, 2000; Vora, 2008, 2012).

In this article, we build on emerging research on how the Indian reproductive industry expands into unregulated markets in East Africa, with a focus on Kenya. While relying mainly on secondary sources, previous research and print media reports, we also draw on a scoping review of web pages marketing ART services in Kenya (conducted between August and September 2022), and on interviews with ART services providers in India [during ethnographic fieldwork in Chennai, Bangalore (South India), Delhi (North India) and Gujarat (West India) - between 2019 and 2023]. We suggest that this expansion to Kenya is productive to view through the lens of the colonial histories of the two countries. For instance, the Indian diaspora, occupying a prominent economic position in contemporary Kenyan society which provides the infrastructure for business expansions, is a legacy of colonial labor migration patterns. More specifically, it can be traced back to the economic dominance of Indian merchants in the British East Africa Protectorate, and Indians being deployed as indentured labor for railway constructionand recruited as clerks into the colonial administration. In these positions, Indians facilitated the imperial mission by occupying an intermediary position between White British colonizers and Black indigenous Africans in the colonial racial hierarchy (Aiyar, 2015).

### The politics of reproductive labor

Recent scholarly interventions, theorize surrogacy as a form of labor, which is both gendered and racialized. Kalindi Vora sees it as a new form of reproductive labor: biological labor – a concept that designates the commodification of the biological capacities of the body such as gestating the embryo (Vora, 2009, 268). It is also a form of affective labor, that is, work involved in caring for others (Vora, 2008). This conceptual move is important, since affective and biological labor is excluded both from the classical Marxist notion of productive labor and from the feminist materialist analysis, which otherwise has usefully pointed out the historical differentiation between productive and reproductive labor (Vora, 2012, 683). Furthermore, the processes of invisibility and devaluation are reinforced by new reproductive technologies and Western understandings of the body. The distinction between gestational carrier and commissioning parent is a case in point, mirroring the separation and hierarchical relation between the physiological aspects and the social aspects of human reproduction. The result is a view of surrogates as the mere bodies in which the genetic material is matured into babies (Vora, 2015).

In our work on the politics of globalized reproduction, we have primarily studied commercial surrogacy in India. Scholars within this field have argued for the centrality of colonialism to understand the global success of Indian surrogacy. More specifically, this work has singled out the recourse to universalizing (ethnocentric) concepts and an associated uneven distribution of humanity, as a critical condition which elucidates why some bodies and not others are seen as the possible sources of commodification (Vora, 2009, 2015) We have drawn on Alys Weinbaum’s notion of the “imperial/colonial episteme” (Weinbaum, 2019, 59) – which posits racialization as a mechanism through which extraction and commodification is facilitated [also see Collins (1990)] – to explore the legacies of colonialism in contemporary practices of globalized reproduction, with a specific focus on transnational adoption and surrogacy (Gondouin and Thapar-Björkert, 2022). By freighting in concepts of racial medicalization, Indian surrogates become perceived as sites of risk and danger to the fetus they gestate (Deomampo, 2016). This mode of thought illustrates a colonial episteme according to which the body is commodified through fragmentation and manifested through the myth of the empty womb as separated from the self and the rest of the body and therefore possible to ‘rent’. Such a construction implies “instituting artificial and rigid lines of separation between the Indian surrogate’s womb and the rest of her body […] between the biological and the affective,” pushing surrogates to “completely disengage from several critical aspects of their identities” (Banerjee, 2014, 124). We understand this violent imposition to be made possible by the coloniality of power: through not recognizing the surrogate as fully human. While it can be argued that processes of fragmentation and commodification are mobilized in all commercial surrogacy arrangements, we suggest that some bodies are more easily subjected to these processes. This, however, does not mean that surrogates passively accept the dehumanizing processes that gestational contract pregnancy may be said to involve. Indeed, in ethnographic accounts such as those detailed by Amrita Pande (2014), Indian surrogates resist being positioned as disposable vessels by claiming kinship through the labor of gestation and the sharing of bodily fluids (blood) that it entails, a claim that challenges existing biological/genetic conceptualizations of kinship. Furthermore, by asserting that the blood of the gestating body not only nourishes the fetus but also shapes its identity, understandings of patriliny and patrilocal kinship models in India are complicated.

### Locating Indian politics on surrogacy

We situate the Indian reproductive industry within a history of population control and coercive reproductive policies targeting marginalized communities. The Indian state was the first in the world to initiate an official population control programme in 1952, a programme shaped by public and private organizations (such as USAID, UNFPA, the Population Council) together with training of administrators and students from formerly colonized countries (Harkavy, 1995). Within the parameters of the Nehruvian policy of modernization post 1947, a selective “appropriation of modernity” and a parallel identification of a certain caste and class of women as the upholders of Indian culture, had important implications for shaping public debates on reproductive control (Chatterjee and Riley, 2001, 820). Influenced by pre-independence contacts with pro-Malthusian British lobby and the Planned Parenthood founder Margaret Sanger, the Nehruvian government in the 1950s allowed organizations such as Population Council, the Ford Foundation and the International Planned Parenthood to administer controlling the population of the Dalits (Zubrin, 2012). Thus Nehruvian democratic socialism and the strong drive for national reconstruction recognized the importance of population control together with economic regeneration. Despite historic wariness of foreign influence, Indian medical professionals and Indian government officials expressed an interest in adopting Western reproductive technologies, at first through controversial Norplant and Depo provera contraceptives followed by coercive sterilization programs (Narayanan, 2011; Hartmann and Rao, 2015). While our intention is not to draw any direct links between Nehruvian politics and commercial surrogacy, we wish to highlight that conversations around reproduction and reproductive labor have a historical precedent, though largely framed through the lens of modernity and expectations of Western donors.

As Amrita Pande (2014) points out, an aggressively pro- population control state becoming a global hub for ART procedures is a noteworthy paradox. The bodies of Dalit and Muslim women in India, formerly seen as “waste” and their reproduction as something to be “controlled,” both by the Indian state and policy makers in the Global World (Rao, 2004; Rao, 2010), become sites of profit generation in the contemporary reproductive market. Within Prime Minister Narendra Modi’s nationalist ideology, upper class and caste Hindu women are tasked with the responsibility of ‘reproducing’ a Hindu nation’ [see Sharma, 2023] to allay the risk of being outnumbered by minority populations, especially Muslims. In our previous work, we have addressed the division of labor within India, structured by hierarchies of caste, for instance how low caste women are tolerated as surrogates while less desired as egg donors, or paid less because of their caste identity (Gondouin et al., 2020, 2022).

Thus, historically, −population control policies and a strong bias toward preventive health care have dominated state programs, although a growing recognition in official state policy of infertility as a legitimate reproductive health problem has been noted (Bharadwaj, 2016, 109; Parry and Ghosal, 2022). The highly specialized sector in assisted conception in India is part of the country’s private healthcare sector (Deepa et al., 2013; Bharadwaj, 2016; Rao, 2016) that has responded to the priorities of state policies by providing curative care, including infertility treatment. The private health care sector has been supported by a range of policy measures, including land grants, reduction in import duties on high-technological medical products and state recognition of medical care as an industry, allowing long-term capital from financial institutions (Deepa et al., 2013; Bharadwaj, 2016). In addition, the Indian state’s investment in training of medical personnel has created a supply of doctors and other medical personnel available for the private sector (Vora, 2015; Bharadwaj, 2016). The Indian Council of Medical Research (ICMR) estimates that between 2005 and 2012, the number of IVF clinics and the generated revenue of IVF and associated technologies had multiplied five times.

Interestingly, the government of India was early to encourage research on reproductive technologies. The introduction of IVF and the birth of the first test tube baby in India in 1978 was the product of a collaboration between the Indian Council for Medical Research (ICMR) and a public hospital in Mumbai, justified as a way to improving knowledge on how to control human fertility by mastering human infertility and the idea that sterilization might be easier to accept if future needs to reproduce could be met by IVF (Rao, 2016; Parry and Ghosal, 2022). However, by the end of the following decade, infertility medicine had been overtaken by private actors who were able to reap the fruits from state-funded research (Bharadwaj, 2016).

### India following the surrogacy (regulation) bill 2020

Regulations of Indian surrogacy have been put in place progressively in response to public debate and the biopolitical agenda of the Indian state. In 2013, single and gay parents were excluded from the market, and in 2016, the Indian Surrogacy (Regulation) Bill was approved and passed as law in December 2021, restricting surrogacy to altruistic arrangements for heterosexual Indian couples with documented infertility. Feminists have criticized the 2021 Act for increasing the vulnerability and potential exploitation of surrogates (Rudrappa, 2018a). Emerging research indicates that altruistic surrogacy in India still entails an instrumentalization of women’s reproductive capacities for securing their families futures, while simultaneously making the terms of their labor more insecure and removing all state responsibility (Hibino, 2023). Furthermore, the new law still sustains the reproductive bioeconomies in India (Pande, 2020), since all forms of surrogacy require an array of procedures and services such as embryo freezing services, fertility consultations, hormonal drugs and the infrastructure of the IVF labs for fertilization and freezing.

The law is particularly criticized for leaving the provision of oocytes unregulated, even though India has the world’s largest egg donation industry within a global *in-vitro* fertilization market estimated to 15 billion USD with an annual growth rate of 10% (Grand View Research 2018). From specializing in babies “Made in India,” India is now emerging as a hub for” pre-conception assemblage” (“Make in India”), where eggs and sperms are assembled into embryos, frozen and/or exported for gestation in women in countries with no surrogacy regulations” (Pande, 2020, 2). In other words, India’s role in the global” reproductive assembly line” (Rudrappa, 2010) has changed in accordance with the country’s shifting legal and economic policies.

In conjunction with this, Indian clinics are relocating to other parts of the world; a pattern known from previous restrictions such as the banning of gay parents, when Indian law was evaded by moving Indian surrogates to Nepal to carry their pregnancies to term, thus simultaneously evading the Nepalese law which prohibited Nepalese women from working as surrogates. Similarly, the subsequent banning of foreign clients made Laos and Cambodia new destinations operated by Indian clinics before they also banned surrogacy (Mitra et al., 2018). Relocating certain procedures abroad allows the industry to continue to employ traveling surrogates and egg donors commercially, which positions surrogates in precarious working environments that are similar to the insecure working conditions of other migrant female care workers from the global South (Mitra et al., 2018; Pande, 2020).

In the present, Africa is emerging as one of several new global hubs for commercial surrogacy, with major Indian clinics expanding on the continent (Parry and Ghosal, 2022). ARTs are currently booming in lower middle-income countries such as Nigeria, Kenya, Ghana and South Africa, but emerging research also indicates its spread to low income countries like Uganda, Mali, Burkina Faso, Sudan and Rwanda (Gerrits, 2016; Hörbst, 2016; Hörbst and Gerrits, 2016; Gerrits, 2018).

### Colonial entanglements

Our inquiry into the expansion of the Indian ART industry to Kenya will seek to locate this contemporary development against the backdrop of the historical economic and political interactions between these countries, including the differentiated positioning of, and close relationship between, India and Kenya during colonialism and in the post-colonial era. Indian trade involvement in East Africa has a long pre-colonial history, emanating with Indian sailors traversing the Indian Ocean with their dhows over thousands of years ago. In mid 19th century, Indian businessmen started setting up trading posts along the shoreline of East Africa, and soon came to control the bulk of the trade in the region, well into the interior (of Kenya and Uganda), where the Indian rupee remained the key medium of exchange until the early 1920s (Blyth, 2003; Metcalf, 2007; Aiyar, 2015).

High representatives of the British colonial administration initially encouraged Indian control of the economy and trade in East Africa – with Sir Harry Johnston, Commissioner of British Central Africa and Special Commissioner in Uganda (1899–1901) envisioning the region as the” America of the Hindu” – a territory fit for colonization by India (Blyth, 2003; Metcalf, 2007). Since Indians occupied a middle-position in the colonial racial hierarchy, they were, in the imaginations of Johnston and many of his compatriots, suited to act as intermediaries between white Europeans and black Africans. This included engaging in the education and ‘civilization’ of black Africans, and bringing ‘modernity’ in the form of trade, while simultaneously carrying out work deemed too menial for white Europeans, and being confined to geographical locations regarded as unsuitable to white people (Blyth, 2003; Metcalf, 2007; Aiyar, 2015). While the recurring proposals that India be given the opportunity to colonize East Africa were turned down by the metropole – the British East Africa Protectorate proclaimed in 1895 (roughly encompassing present-day Kenya) became a crown colony in 1920 – the British empire relied heavily on the presence and economic know-how of Indian tradesmen in order to establish colonial rule throughout the region (Aiyar, 2015). Indeed, in the words of Thomas R. Metcalf (2007, 188), East Africa was in the late 1890s and early 1900s” a colony as much of British India as of Britain itself.” For instance, the British colonial flagship project of constructing a railway line from the Kenyan coast to Uganda was in its entirety a product of British India: from the indentured Indian laborers carrying out the bulk of the manual work – a total of 32,000 Indians worked on the railway between 1896 and 1902 – to the British managers and administrative and technical staff who had previously worked in British India, to the locomotives and indeed the rules and regulations structuring railway operations (Metcalf, 2007). A substantial number of Indians furthermore migrated to the region to engage in small-scale trade and artisanal work, and to take up positions as clerks, policemen and supervisors of African subordinates in the colonial administration (Metcalf, 2007; Aiyar, 2015). Many of the indentured laborers, skilled workers and businessmen returned permanently to India after a few years, while others remained but regularly traveled to India for business purposes, higher education, marriage and childbirth (Aiyar, 2015).

While Indians undoubtedly occupied an intermediary position in the British East African Protectorate, whether as manual laborers with higher qualified and better paid positions compared to their African compatriots, albeit much lower in rank than the white British, or as tradespersons who were at the same time, unlike white settlers, prohibited from purchasing agricultural land, the material conditions, access to social capital and political affiliations among Indians in pre-independence Kenya were indeed highly diverse and complex (Metcalf, 2007; Aiyar, 2015). Whereas many Indians actively sought to place themselves above Africans, and aspired to parity with the white British settlers, others advocated solidarity among non-white populations. For instance, renowned trade union leader Makhan Singh spoke out in support of African workers going on strike for better pay, and in the 1930s, after having succeeded in pushing Indian businessmen to raise the pay for Indian laborers, opened up the East African Trade Union to membership from Africans (Aiyar, 2015).

As discussed by Aiyar (2015, 13), how the Indian administration and its diaspora related to Kenya and vice versa has furthermore fluctuated over time, corresponding with (post-) colonial power hierarchies and reflecting a “simultaneous coexistence of solidarity and friction”. This includes India and its diasporic community’s claims of equality with white Europeans in the early 20th century, drawing on successful Indian business expansion into the interior of the British East African Protectorate, to cross-racial solidarity against the British Empire in the late 1940s and early 1950s, to highlighting Indian businesses’ contribution to economic development in Kenya as an argument for nationalist claims to the newly liberated country as a homeland. At the time of Kenya’s independence in 1963, Indians constituted 2 % of the Kenyan population, and nearly one third of the residents of Nairobi, making up a substantial share of the petty bourgeoisie (Aiyar, 2015). However, Indians did not automatically become citizens of Kenya, but had to file an application – a process that many either lacked the financial resources for, or were reluctant to initiate, as class- and racial tension was mounting in the years following independence. Drawing on a “discourse of racial majoritarianism” (Aiyar, 2015, 269), the government responded to the majority population’s frustration at the slow pace of socio-economic improvement by directing blame toward Indians, who were positioned as aliens in the new nation and unscrupulous business people, despite the active role played by some Indians in Kenya’s struggle for independence. When new legislation was introduced that revoked permanent residency for non-citizens while curbing business opportunities for non-African traders, and Britain shortly thereafter threatened to restrict in-migration for Indians with a British passport, this sparked a voluntary exodus of Indian Kenyans to Britain (Aiyar, 2015). While more than half of the Indian Kenyans had left the country 15 years after independence, there has since been a steady flow of Indian migrants arriving to settle temporarily in the country, working as skilled labor in software development, medicine, private businesses or other sectors (Modi, 2010). Today, Kenya is home to the largest Indian community in East Africa, with about 80,000 Indians of whom about 60,000 are categorized as People of Indian Origin (PIO) and 20,000 as Indian citizens.^1^

### Contemporary relations

India has entertained economic relations with Africa since the post-independence years, with the liberalization of India’s restrictive policies on domestic companies’ investments abroad in the 1990s giving momentum to trade and investment relations between India and East Africa, further intensified in the early 2000s. In 2022, India was the second largest investor after China in the region, with Kenya ranking as its main market and India being one of the main trading partners of Kenya.^2^ Singled out as pivotal for socio-economic growth, the healthcare sector has been the focus of all major initiatives from the Indian State toward Africa since the early 2000s (Saint-Mézard and Nicolas, 2022). India’s implication is multifaceted, involving both private and public stakeholders, and ranging from provision of drugs, treatments, technology and skilled workers, contributions to local capacity-building and investments in healthcare infrastructure to establishing Indian hospital chains in the region, primarily in Nairobi (Saint-Mézard and Nicolas, 2022). It also includes initiatives such as the Pan African e-Network Program (PANe-NP), launched in 2009, aiming to give hospitals in the African Union access to the expertise of Indian practitioners in Indian medical institutes through a satellite and fiber-optic network, in particular in the fields of tele-education and telemedicine. This venture, with an annual budget of USD 200 million, enabled renowned infertility hospitals such as Rotunda and Apollo to establish themselves in the region, and was until 2017 entirely funded by the government of India. The program was refurbished in 2019 as e-VidyaBharti (Tele-education) and e-ArogyaBharti (Tele-medicine), aiming to “provide free tele-education to 4,000 African students each year for five years and to continue medical education for 1,000 African doctors, paramedical staff, and nurses.” (Saint-Mézard and Nicolas, 2022, 25).^3^

Furthermore, India has become a preferred destination for medical tourism from the region. Middle class East Africans travel to clinics in India for affordable specialist therapies such as cardiac and cancer treatments. In Kenya, India has become the number one destination for overseas medical services (Saint-Mézard and Nicolas, 2022). Indian exports to East Africa reflect demand and inadequate domestic supplies, but the dependency that it creates has also become a matter of tension. This became manifest during the Covid-19 pandemic regarding supplies of vaccines and air travel restrictions. Medical tourism in particular has been criticized for preventing the development of local facilities and competence (Saint-Mézard and Nicolas, 2022).

### Dispensable bodies

If one browses the web pages of companies in Kenya that offer ART services, including surrogacy, the interconnectedness with India soon becomes apparent.^4^ One company operates as a subsidiary of an Indian IVF hospital chain, others are run by Indian medical doctors or feature staff members mainly from India and Nepal, having undergone their medical training in India. Some company web pages use the Indian internet country code or list Indian contact numbers that prospective clients may call for advice. Kenya and neighboring Uganda, where the ART industry has also begun to expand, currently lacks legislation regulating the rights and obligations of ART providers, gamete donors, surrogates and patients – with attempts to introduce such legislation having been met with opposition primarily from ultra-conservative religious groups, opposing ARTs in its entirety. The industry thus relies on self-regulation and adherence to general medical principles and protocols, opening up for the presence of unscrupulous actors alongside more established clinics that seek to offer quality services, albeit operating under additional financial, legislative and infrastructural challenges compared to in India or Europe (Nampewo, 2021). In this context, the practice of commercial surrogacy and the labor conditions of women engaged as surrogates is particularly contentious. The construction of Kenyan surrogates as first and foremost cheap labor is illustrated in the following quote from the web page of one service provider: “The cost of Surrogacy in Kenya is one-fifth of the other well-developed countries and this is the major reason why every year number of patients [*sic*] prefer to travel down to Kenya for their surrogacy procedure to fulfill their dream of having an own baby.”^5^ This quote is exemplary of the shifting geo-political positionalities of countries within the ART global circuits. It resonates with the pre-ban designation of India as offering “First World medicine at Third World prices” (Rudrappa and Collins, 2015, 953). Furthermore, unlike in India, several ART providers in Kenya market themselves as offering both traditional and gestational (commercial) surrogacy. While traditional surrogacy is a less complicated medical procedure, most contemporary providers focus on gestational surrogacy due to the emotional and legal complexities of the former.^6^ Interestingly, it is the lack of genetic connection to the person carrying the pregnancy to term which is often emphasized in accounts of the success of outsourcing of surrogacy to the global South. While exact figures of the size of the surrogacy market in Kenya are not available, estimates suggest it is a fairly new and small market. Yet, with infertility rates in sub-Saharan Africa being among the highest in the world, the scarcity of IVF clinics, and neglect of infertility by the public healthcare systems (Inhorn and Patrizio, 2015; Hörbst and Gerrits, 2016), makes the region highly attractive area for investors. Parry and Ghosal (2022) claim that Indian ART providers have in recent years taken over the role earlier played by European colonial powers, notably the UK and Germany, that up until recently dominated the markets in Africa. This, they suggest, mirrors “the historical dynamics of colonial expansion, in which Indian elites worked alongside the colonial state, acting as a comprador class there to represent and manage the colonizers’ interests in Kenya, Nigeria, or Ghana, before finally taking control of the market themselves.” (Parry and Ghosal, 2022, 17).

According to media reports, surrogates in this region are highly vulnerable. In 2020 and 2021, investigative journalist Lepapa (2021a,b) went undercover as a prospective surrogate mother and a commissioning parent with different agencies, interrogating allegations of exploitation and fraud in the surrogacy industry. She documented cases of surrogates being made to abort without their consent in the final stages of pregnancy, of surrogates being pressured to travel to foreign countries to give birth at the threat of having their compensation withheld, of surrogates being paid less than what they had been promised and denied the right to keep the contracts that they signed, patterns that have been documented in India as well. One of the companies featured in Lepapa’s (2021a,b) exposé is an offshoot of an Australian company founded by an Indian businessman, who had operated in India until the 2013 ban on surrogacy for foreign same-sex couples and single parents, and then moved on to Nepal and Cambodia before starting up operations in Kenya. Reports of Kenyan surrogates traveling to India for embryo implantation and returning to Kenya to give birth and hand over the child to prospective parents – in this way circumventing regulations in the two countries – have featured in media reports, and also emerged in Rudrappa’s (2018b) research on the Indian ART industry. Rudrappa describes how Kenyan traveling surrogate mothers “become analogous to shipping containers” acting as cross-border “cargo carriers of life—life that *a priori* belong to clients” (Rudrappa, 2018b, 1093). Creating and exploiting such unregulated “in-between-spaces” is identified as a trademark of contemporary racial capitalism and its co-articulation with bordering practices and migratory regimes (Bhattacharyya, 2023).

In attempting to attract foreign clientele, some IVF service providers in both Kenya and Uganda furthermore marketed themselves either in text or visual representation as accommodating non-heterosexual commissioning parents from overseas. This despite the criminalization of same sex intimacy in both countries, the prohibition of adoption by sexual minorities, and increased restrictions on international adoptions from Kenya in 2019.^7^ A text on the site “Surrogate Mother Kenya,” which lists an Indian contact number, curiously describes the current lack of regulation in Kenya as an act of love and kindness by the Kenyan government, a gesture which is equally welcoming to heterosexual and same-sex couples:

The government of Kenya is very kind, and hence they have no legal Surrogacy Law in Kenya. Kenya is a country where couples of same-sex or different-sex can travel for their surrogacy procedure. /…/ The government of Kenya understands that surrogacy is just a procedure which helps the childless couples to enjoy the parenthood happily. This is the reason that there is no legal Surrogacy Law in Kenya.^8^

It is symptomatic how the marketing of the loving, kind, welcoming (and affordable) surrogacy market in Kenya, in the wake of the ban in India, completely silences the presence of black surrogate mothers and the conditions under which they labor, while wealthy, foreign commissioning parents are depicted as worthy of the prospect of happiness and parental fulfillment.

## Conclusion

In this article we explore the emergence of extractive economies following the Indian ban on commercial surrogacy. We map the restructuring of the Indian ART industry into East Africa (drawing on Kenya as a case study), where access to ART services remains scarce despite high infertility rates, often caused by preventable diseases (Inhorn and Patrizio, 2015). The present expansion of the ART sector in East Africa, which in the past decade has come to be dominated by Indian companies and medical staff in Kenya and elsewhere on the continent (Parry and Ghosal, 2022), is fueled by an increasing demand for ART services and an ART industry post ban in search of new opportunities and locations. Importantly, we suggest that reproductive patterns of exchange have been facilitated through colonial and postcolonial links between India and East Africa which has created a sizable, and comparatively wealthy, South Asian (mainly Gujarati and Punjabi) minority in Kenya (Herzig, 2010). This linkage was clearly stated in an interview with a gynecologist who runs a private women’s hospital in Gujarat, “Patels [land-owning farmers who later established relationships with British East India Company, as businessmen and merchants] want pregnancy immediately. Hence, if they do not conceive very soon, they seek the doctor. Because of historic links with East Africa we see many families coming to Gujarat for fertility treatments. Families in Gujarat are often in consultation with kith and kin in East Africa.” (Interview conducted by third author, August 2023). The division of reproductive labor in this emerging sector can also be productively understood through the prism of stratifications pertaining to hierarchies of race and labor. As such, it informs the positionality of these necessary, yet expendable and disposable racialized reproductive workers – who, in the words of Clarisse Burden-Stelly (2020), produce (surplus) value minus worth – shaping both the particularity of their labor (reduced to their bodily functions through biological labor) and the conditions under which this work is performed.

## Author contributions

All authors listed have made a substantial, direct, and intellectual contribution to the work and approved it for publication.

## Footnotes

^1^MEA, 2022, *Population of Overseas Indians*, available at: http://mea.gov.in.

^2^See, e.g., https://mea.gov.in/Portal/ForeignRelation/India-Kenya_sep_2019.pdf.

^3^Memorandum of Understanding between the Indian Council of Medical Research and the African Union on India-Africa Health Sciences Cooperation: https://main.icmr.nic.in.

^4^The review of web pages referred to throughout this section was conducted in August and September 2022.

^5^https://kenyaivf.com/surrogacy-kenya/, accessed September 14, 2022.

^6^https://surrogate.com/about-surrogacy/types-of-surrogacy/traditional-vs-gestational-surrogacy-whats-best-for-my-family/; https://southernsurrogacy.com/, accessed September 30, 2022.

^7^Since surrogacy is unregulated in Kenya, commissioning parents would need to go through the courts in either seeking to adopt a child born of a surrogate mother, or apply for legal custody and parental orders. However, international adoptions were banned in 2019, and, while exemptions can be made in “special instances,” sole applicants, unmarried couples, “homosexuals” and “sole foreign males” are never permitted to adopt according to the current legislation (Lepapa, 2021a,b).

^8^https://surrogatemotherkenya.com/, accessed September 14, 2022.
