# Corrigendum: Chains of extraction: shifting bioeconomies in India and East Africa

**DOI:** 10.3389/fsoc.2025.1532386

**Published:** 2025-02-18

**Authors:** Johanna Gondouin, Åsa Eriksson, Suruchi Thapar-Björkert

**Affiliations:** ^1^Department of Ethnology, History of Religions and Gender Studies, Stockholm University and Mångkulturellt Centrum, Stockholm, Sweden; ^2^Department of Government, Uppsala University, Uppsala, Sweden

**Keywords:** India, East Africa (Kenya), surrogacy, biocapitalism, colonial histories, globalization - economic development

In the published article, there was an error. A citation was accidentally inserted, and there was a mistake regarding the year that the bill was approved as law.

A correction has been made to the section *India following the surrogacy (regulation) bill 2020*, paragraph 1. This sentence previously stated:

“In Deepa et al., 2013, single and gay parents were excluded from the market, and in 2016, the Indian Surrogacy (Regulation) Bill was approved and passed as law in December 2020, restricting surrogacy to altruistic arrangements for heterosexual Indian couples with documented infertility.”

The corrected sentence appears below:

“In 2013, single and gay parents were excluded from the market, and in 2016, the Indian Surrogacy (Regulation) Bill was approved and passed as law in December 2021, restricting surrogacy to altruistic arrangements for heterosexual Indian couples with documented infertility.”

In the published article, there was an error. A mistake regarding the year of the act.

A correction has been made to the section *India following the surrogacy (regulation) bill 2020*, paragraph 1. This sentence previously stated:

“Feminists have criticized the 2020 Act for increasing the vulnerability and potential exploitation of surrogates (Rudrappa, 2018a).”

The corrected sentence appears below:

“Feminists have criticized the 2021 Act for increasing the vulnerability and potential exploitation of surrogates (Rudrappa, 2018a).”

The authors apologize for these errors and state that this does not change the scientific conclusions of the article in any way. The original article has been updated.

